# ROMI: A Real-Time Optical Digit Recognition Embedded System for Monitoring Patients in Intensive Care Units

**DOI:** 10.3390/s23020638

**Published:** 2023-01-05

**Authors:** Sanghoon Jeon, Byuk Sung Ko, Sang Hyuk Son

**Affiliations:** 1 Department of Emergency Medicine, College of Medicine, Hanyang University, Seoul 04763, Republic of Korea; 2 Department of Electrical Engineering and Computer Science, Daegu Gyeongbuk Institute of Science and Technology (DGIST), Daegu 42988, Republic of Korea

**Keywords:** optical digit recognition, real-time monitoring, medical devices, embedded systems, intensive care units

## Abstract

With advances in the Internet of Things, patients in intensive care units are constantly monitored to expedite emergencies. Due to the COVID-19 pandemic, non-face-to-face monitoring has been required for the safety of patients and medical staff. A control center monitors the vital signs of patients in ICUs. However, some medical devices, such as ventilators and infusion pumps, operate in a standalone fashion without communication capabilities, requiring medical staff to check them manually. One promising solution is to use a robotic system with a camera. We propose a real-time optical digit recognition embedded system called ROMI. ROMI is a mobile robot that monitors patients by recognizing digits displayed on LCD screens of medical devices in real time. ROMI consists of three main functions for recognizing digits: digit localization, digit classification, and digit annotation. We developed ROMI by using Matlab Simulink, and the maximum digit recognition performance was 0.989 mAP on alexnet. The developed system was deployed on NVIDIA GPU embedded platforms: Jetson Nano, Jetson Xavier NX, and Jetson AGX Xavier. We also created a benchmark by evaluating the runtime performance by considering ten pre-trained CNN models and three NVIDIA GPU platforms. We expect that ROMI will support medical staff with non-face-to-face monitoring in ICUs, enabling more effective and prompt patient care.

## 1. Introduction

Critical care is the process of medical care for patients with potentially life-threatening injuries and illnesses [1]. Critical care usually takes place in intensive care units (ICUs). A specially trained team continuously monitors the vital signs of patients and provides immediate critical care when the patients are at risk [2]. In other words, they are responsible for recognizing early signs of deterioration in patients and responding appropriately to prevent subsequent events and reduce patient mortality [3].

Subtle changes in vital signs, such as respiratory rate, blood pressure, heart rate, body temperature, and oxygen saturation, are early warning signs of clinical deterioration [4]. A noninvasive hemodynamic monitoring device is commonly used to monitor the vital signs of patients in ICUs [5]. Modern hemodynamic monitoring devices, such as the IntelliVue and IntelliBridge systems from Philips, have built-in connectivity capabilities that allow for seamless real-time data transfer to hospitals’ electronic medical record (EMR) systems [6]. However, only one-third of hospitals have interfaces to connect bedside devices, such as infusion pumps, ventilators, and hemodynamic machines, to the EMR system, thus necessitating manual inspections by medical staff [7]. This is because the functional interoperability of medical devices and their integration with the EMR system are limited [8]. For example, the Philips IntelliBridge system is developed for data integration and collects data according to the standard data protocol Health Level 7 (HL7) [9]. However, the system was developed for data exchange among Philips products and is not compatible with devices from other manufacturers [10]. In addition, it is difficult to use the system in many hospitals due to additional costs, such as the product price, installation, operation, and maintenance.

With the development of Internet of Things (IoT) technology, the IoT is expected to improve the quality of medical services, patient safety, management efficiency, and patient-centered medical services [11]. The basic concept of the IoT is to connect anything or everything that can be connected to the internet [12]. The IoT has demonstrated its potential to deliver quality healthcare, improve patient safety, reduce healthcare costs, and improve healthcare access in remote locations by connecting various medical devices, sensors, and healthcare professionals [13]. For example, *VitalPAD* was developed to improve the efficiency of clinical decision making, communication, and patient safety by combining information from multiple monitoring and treatment devices in a mobile application [14]. *HEAL* was proposed to automatically and unobtrusively monitor events and activities in an ICU room by using multimodal distributed cameras [15]. *SensableCare* is an alert system that delivers a timely alert to a nurse via a mobile device when it detects that a patient has moved out of bed [16].

During the COVID-19 pandemic, we experienced a serious threat to public health worldwide. Medical staff are in direct contact with patients on the front line and are exposed to risks such as infection, lack of sleep, and overwork [17]. Developing countries, including Bangladesh, faced unprecedented challenges, such as medical staff and equipment shortages, lack of personal protective equipment, fear of infection, and social exclusion [18]. A lesson from the COVID-19 outbreak was that the virus can spread rapidly between patients and medical personnel, increasing the risk of cross-contamination [19]. IoT-based remote control of medical devices in ICUs [20] or remote monitoring of COVID-19 patients [21] can be an effective solution for responding to future infectious diseases in non-face-to-face forms.

Robots could be a promising alternative in epidemic outbreaks, such as that of COVID-19. Almost every industry faced many difficulties during the COVID-19 pandemic, but the acceptability and opportunities of robotic systems have increased [22]. Robotic systems could be used for many different purposes, such as diagnosis, screening, disinfection, surgery, and telehealth during COVID-19. The primary role of medical robots in clinical settings is to prevent the spread of infection among frontline medical personnel by minimizing human-to-human contact and isolating direct exposure to disease [23,24].

**Figure 1:**
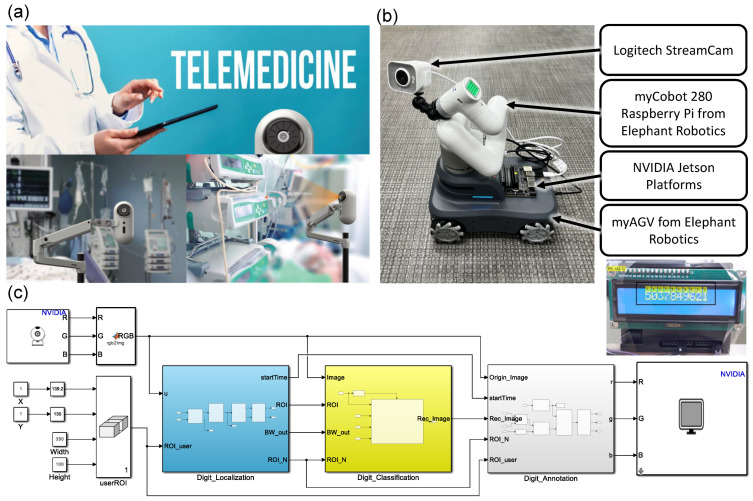
A real-time optical digit recognition embedded system for monitoring patients in ICUs. (**a**) A concept of ROMI for monitoring patients in ICUs. (**b**) Hardware prototype of ROMI. (**c**) System configuration of ROMI in Matlab Simulink.

To effectively monitor patients in ICUs and similar facilities, such as quarantine centers, we propose a real-time optical digit recognition embedded system for monitoring patients in intensive care units (ROMI). ROMI is embedded in a mobile robot and serves to monitor patients in ICUs based on robotics and IoT concepts, as shown in Figure 1a. For example, a mobile robot approaches a medical device, controls a robot arm equipped with a camera, recognizes the digits on the display, and transmits the recognized data to a control center in real time to monitor patients in ICUs.

The core algorithm of ROMI is optical characteristic recognition (OCR). OCR is used in various applications, such as converting handwriting into editable text, identifying vehicle license plates, and converting scanned/printed documents or natural scene images into text [25]. Several algorithms were developed over a long time, such as open OCR algorithms—tesseract OCR [26], easyOCR [27], and keras-OCR [28]. These algorithms are good open-source APIs that are freely available. However, there is no such thing as a perfect OCR algorithm, especially in real-world conditions. To the best of our knowledge, not all OCR algorithms are perfect, as they need to be slightly modified according to new conditions to ensure the algorithm’s performance.

Due to the challenges in recognizing digits in the real world, it is necessary to supplement the algorithm through initial calibration and data collection for each medical device. In this work, we propose a general process for developing a real-time digit recognition model with transfer learning and applying it to embedded systems by using Matlab Simulink as a proof-of-concept study. We evaluated the recognition performance to select the best model out of ten pre-trained convolutional neural network (CNN) models. We also investigated the runtime performance according to the NVIDIA Jetson GPU platforms. This work makes the following contributions:

- We propose a real-time digit recognition embedded system called ROMI. ROMI consists of three subsystems, i.e., digit localization, digit classification, and digit annotation. The subsystems of ROMI were developed by using Matlab Simulink. In this work, we demonstrate the entire process for developing ROMI—from data acquisition and model development to embedded system deployment—as a proof-of-concept study.
- Not all OCR algorithms are perfect under real-world conditions. Implementing deep learning (DL) models in the real world requires calibration, which involves collecting new training datasets and training/fine-tuning the models. We used data augmentation on a small training dataset to easily and quickly calibrate DL models in the initial setup.
- We retrained ten pre-trained CNN models to develop a digit recognition model with transfer learning. We then selected the best DL model, i.e., alexnet, through a comprehensive recognition performance evaluation.
- We created a benchmark for ROMI by deploying ten trained DL models on three NVIDIA graphics processing unit (GPU) embedded platforms to analyze the runtime performance.

The rest of this paper is organized as follows. Section 2 discusses OCR-related research. Section 3 describes the digit dataset, subsystems of ROMI, and embedded hardware platforms, respectively. Section 4 shows the evaluation results for the digit recognition and runtime performance on the NVIDIA GPU platforms. Section 5 discusses the limitations of this work and future work. Finally, this paper concludes in Section 6.

## 2. Related Work

This section addresses OCR research for seven-segment display digit recognition applications. We also discuss the significance of the OCR technique in ICUs for integrating ICU data into EMRs.

OCR research has been studied for a long time. However, when using OCR in real life, it performs worse than expected. Tesseract OCR is a popular OCR model developed by Google, but it has trouble reading seven-segment displays correctly and only reads plain text on pages well [29]. Discontinuities in digit representation on a seven-segment display degrade the performance of tesseract OCR and require appropriate pre-processing [30].

Pre-processing has a significant effect on OCR performance, especially for images obtained from digital cameras [31]. Therefore, many OCR-related studies focus on pre-processing techniques. For example, Kulkarni et al. [32] used pre-processing methods, such as tilt correction, background elimination, and noise filtering, to make clear digit images. Tsiktsiris et al. [33] proposed an adaptive thresholding method for making binarized images according to pixel intensity. The adaptive thresholding method effectively removed artifacts caused by shadows and unwanted reflections from the screen. The HSV color-slicing technique was also used to separate digits from the background by using predefined HSV parameters [34]. Finnegan et al. [35] proposed pre-processing methods, such as maximally stable extremal regions (MSERs), to find the digit regions, and they used rule-based filtering and blob clustering to get rid of the noise around them. Wannachai et al. [36] proposed image processing methods, such as image transformation (rotation and cropping), noise canceling, and a post-processing method (adaptive bound criteria), to improve the accuracy of digit recognition.

To the best of our knowledge, there is no perfect OCR for resolving these artifacts in real-world conditions. The best way to mitigate this issue is to calibrate the OCR in a pre-processing step before using it based on new data. In our system, pre-processing settings, including dataset generation for training DL models, can be easily modified based on the input image properties, making it applicable to many medical devices.

Advances in IoT have enabled a patient-centric approach by monitoring vital signs to more accurately assess patient health and take predictive actions [37]. However, integrating data from medical devices into EMRs is challenging because many medical devices are not originally designed for network connectivity. In addition, some devices with communication capabilities often use proprietary protocols rather than standard protocols, making data unavailable to end users [38]. As part of an effort to monitor various medical data, an integrated system using an open embedded system was developed. For example, Medical Device Dongle (MDD) [39] was developed to enable interoperable medical device connectivity by using a standard protocol. MDD uses RS-232 or USB for data exchange. CodeBlue [40] is an ad hoc sensor network infrastructure for tracking patient status and location in emergency medical care. CodeBlue collects vital signs, such as oxygen saturation (SpO2) and electrocardiogram (ECG) data, via a MICA2 mote equipped with an RS232 data port. OpenICE [41] is an open-source software project for providing interoperability by connecting multiple medical devices. OpenICE uses Beagle-Bone single-board computers to connect medical devices directly and allow them to communicate through a serial port. AlarmNet [42] is designed for long-term monitoring of older adults and monitoring of physiological sensors, such as ECG, pulse, and blood pressure sensors, via MicaZ and Telos Sky motes with RS232 data ports. MEDiSN [43] was developed to provide effective care during disaster events. MEDiSN uses a mote called miTags [44] and collects physiological signals, such as pulse oximetry and pulse rate.

These approaches commonly use serial communication to read data from medical devices with auxiliary devices, such as a mote, a dongle, and an open embedded system. However, data access is only possible when the medical device provides its own data protocol. In addition, some medical devices do not have communication capabilities. More than one hundred medical devices are installed in large hospitals [45], making it physically impossible to use auxiliary devices for each device. To alleviate these limitations, we opt for a vision-based approach for monitoring multiple ICU medical devices by using a robotic system equipped with a camera. Several similar studies have been conducted due to the COVID-19 pandemic. For example, VentConnect [46] was designed to remotely monitor ventilators. VentConnect uses a display interface converter instead of a camera to transmit the ventilator screens directly to a server. This method is restricted to medical devices with display outputs. PACMAN [47] was designed to monitor the oxygen saturation and pulse rate in COVID-19 patients by reading digits on a pulse oximeter. However, only the pulse oximeter was trained for the OCR model of PACMAN. Thus, model calibration, such as image pre-processing, data collection, and model re-training, is required to apply to various medical devices.

We summarized the related studies on medical data monitoring and integration, as shown in Table 1.

## 3. Methods

ROMI was designed to monitor patients in ICUs, as shown in Figure 1a. The main objective of ROMI is to recognize digits on medical device displays. ROMI detects regions of interest (ROIs) in captured images, classifies the digits in those regions, and attaches bounding boxes with digit class labels to the images. ROMI consists of three main subsystems for these functions: digit localization, digit classification, and digit annotation. This section describes the process of designing each subsystem of ROMI.

ROMI is a robotic system consisting of a mobile robot, NVIDIA Jetson GPU platforms, and a robot arm equipped with a camera, as shown in Figure 1b. We used a Logitech StreamCam camera, which was mounted on a robot arm. The Logitech StreamCam could stream and record in full HD 1080p resolution at 60 frames per second (FPS). ROMI used VGA resolution (640 × 480) at 30 FPS considering the operating speed in embedded systems. The robot arm was a myCobot 280 Raspberry Pi from Elephant Robotics [48]. The robot arm was a six-axis robot and had a light weight of 850 g. The payload was 250 g, and the arm span was 280 mm. We used three NVIDIA GPU embedded platforms: NVIDIA Jetson Nano, NVIDIA Jetson Xavier NX, and NVIDIA AGX Xavier. The mobile robot was the myAGV product from Elephant Robotics [49]. The size, weight, and battery life were 311 mm × 236 mm × 130 mm, 3.6 kg, and 1 to 1.5 h, respectively. The SLAM Lidar sensor was also built in for real-time mapping and scanning, obstacle avoidance, and automatic path planning. Both the mobile robot and the robot arm could be programmatically controlled. In this work, we focused on developing a digit recognition embedded system. In future work, we will add the control blocks for the mobile robot and robot arm.

We built ROM by using Matlab Simulink, as shown in Figure 1c. Matlab Simulink is an effective development tool because it provides a graphical programming environment for modeling, simulating, and implementing models directly in embedded systems. The ROMI code created using Matlab Simulink is a key building block for real-time digit recognition, and we opened the code on a personal GitHub site (https://github.com/SanghoonJ/ROMI-digit-recognition.git (accessed on 1 January 2023)).

### 3.1. Proof-of-Concept Study

**Figure 2:**
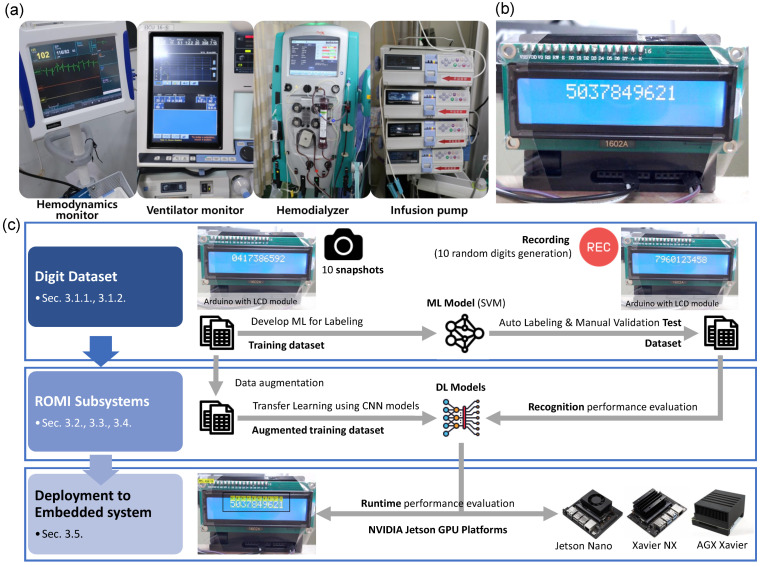
A real-time optical digit recognition embedded system. (**a**) Medical devices in ICUs. (**b**) A proof-of-concept device. (**c**) Flow diagram of the development of ROMI.

There are many types of medical devices for treating critical patients in ICUs, as shown in Figure 2a. Hemodynamic monitors, ventilators, hemodialysis machines, and infusion pumps are examples of typical medical devices used on critically ill patients. The ultimate objective of ROMI is to monitor medical devices in ICUs. Most medical devices consist of liquid crystal display (LCD)-type displays with different font styles, colors, and backgrounds. In our experience, we have empirically confirmed many errors in simply applying open OCR algorithms to the real world. For practical use in real-world medical applications, fine-tuning and calibration of the OCR algorithm are required for each medical device.

A proof-of-concept study is usually an early-stage test to determine the feasibility of an idea. Figure 2b shows an Arduino system equipped with an LCD instead of medical devices. We aimed to recognize the digits displayed on the Arduino as a proof-of-concept study. We programmed the Arduino system with an LCD display to display ten random digits every 10 s on the screen, just like on a medical device. We note that this work focused on developing the general process of a digit recognition embedded system as a proof-of-concept study. A study on digit recognition for actual medical devices in ICUs will be conducted in future work, as it is beyond the scope of this study.

Figure 2c shows the overall flow diagram of the development of ROMI from data acquisition to embedded system deployment. ROMI was designed to improve the efficiency of initial calibration by developing DL models with a small training dataset of ten snapshot images. We first collected ten snapshot images and constructed a training dataset. Then, we trained a machine learning (ML) model by using the training dataset. The trained ML model was used for the automatic labeling of video data. After manually verifying the automatic labeling, we constructed a test dataset, i.e., a semi-automated approach. ROMI used a DL model as a classifier for digit recognition. A data augmentation technique was used to generate the large amount of data required for DL model training, i.e., an augmented training dataset. We trained DL models by using the augmented training dataset. Finally, we evaluated the recognition performance of the trained DL models by using a test dataset. We also deployed the models on the NVIDIA Jetson GPU platforms and evaluated the runtime performance.

#### 3.1.1. Raw Digit Data

We collected two sets of raw data for developing and evaluating ROMI: a training dataset and a test dataset. We constructed two separate datasets by using image data as the training dataset and video data as the test dataset, as shown in Figure 2c. First, we used only ten captured images for the training dataset to reduce the effort of initial data collection and calibration. Since collecting a large amount of training data for deep learning is time-consuming and laborious, we used a method, i.e., data augmentation, to effectively train the deep DL model of ROMI with a small amount of training data. Second, we recorded the LCD screen for about 2 min, and the video data were used for the test dataset. The reason for configuring the test dataset with video data was to evaluate the digit recognition performance in real time.

#### 3.1.2. Dataset Labeling

In this section, we describe the pre-processing of the raw data and the labeling of the data to construct a training dataset and test dataset for ROMI. The labeling task was essential, but tedious and time-consuming. Fortunately, Matlab provided image labeler (*imageLabler*) and video labeler (*videoLabler*) apps to make labeling easier and more efficient. Both apps provided custom automation algorithms for labeling. We applied different automation algorithms separately for the training and test datasets using the image labeler *imageLabler* and video labeler *videoLabler*, respectively.

For the training dataset, we used an automation algorithm (one-class detection) on the image files to create a dataset consisting of data and labels by using the image labeler *imageLabler*. Here, the automation algorithm was configured to detect only one class, i.e., digits. Drawing and modifying ROI regions was time-consuming, but the automation algorithm helped make the labeling task more efficient. After labeling the data with ‘Digit’ by using the automated algorithm, we manually marked the ten sub-labels from ‘Digit 0’ to ‘Digit 9’.

To make the labeling task on the test dataset more efficient, we developed an ML model, i.e., a support vector machine (SVM), by using the training dataset. We trained the SVM model by using the histogram of oriented gradients (HOG) features [50] and implemented it by using the *fitcecoc* function in Matlab. We performed automatic labeling on the test data by using the trained SVM model. Note that we used the SVM model as an ancillary aid in labeling, so we did not evaluate its classification performance. After performing automatic labeling, all datasets used in this work were manually validated, i.e., this was a semi-automated approach.

For the test dataset, we used an automation algorithm (ten classes detection) on the video files to create a dataset consisting of data and labels by using the video labeler *videoLabler*. Then, we manually checked the test dataset consisting of data and labels. In addition, as part of data cleaning, the dataset was excluded while the digits changed on the LCD screen.

### 3.2. Digit Localization (ROMI Subsystem 1)

Digit localization is the first subsystem of ROMI. Depending on the medical device, appropriate pre-processing is required for system performance. Digit localization has two pre-processing steps: the image segmenter and color thresholder. The image segmenter allows the user to select a desired area on the LCD screen for digit recognition. The color thresholder creates a binary image picture to make an identifiable binary image.

#### 3.2.1. Image Segmenter

A large number of monitoring areas increases the computational burden. The first task is to select a region of interest (ROI) on the display of a medical device. In this case, the ROI is a user-defined ROI designating a region where the user wants to recognize a digit number. The user ROI $ROI_{user}$ is defined as a 1 × 4 matrix [vPos, hPos, vSize, hSize]. vPos, hPos, vSize, and hSize indicate the vertical position, horizontal position, vertical size, and horizontal size, respectively, from the top left corner of the image frame.

A binary ROI mask BW is computed by using the poly2mask function in Matlab, which sets pixels inside the polygon to 1 and pixels outside the polygon to 0. Then, by using the binary ROI mask, BW is used in the input image Img, and we transform the non-interest region to zero, as described in Equation (1).

(1) Img(¬BW)=0

#### 3.2.2. Color Thresholder

We used a color thresholder app in Matlab to make a clear binary image. The color thresholder app divided a color image by thresholding the color channels based on different color spaces. We used the hue, saturation, and value (HSV) color space, converted the binary segmentation mask to gray, and, finally, convert it into a binary image.

We performed additional pre-processing to make clear digit images, such as in morphological image processing. There are many methods for morphological image processing [51]. Morphology is a set of image processing operations based on shape. The basic operations are *Dilation* ⊕ and *Erosion* ⊖, and they perform opposite roles. *Dilation* ⊕ makes objects in an image more visible by adding pixels to the boundaries of the objects and filling small holes in the image. For example, lines appear thicker, and filled shapes appear larger. *Erosion* ⊖, on the other hand, removes floating pixels, thin lines, and small holes, leaving only real objects. For example, noise around objects disappears, lines become thinner, and shapes become smaller.

To make a clear digit image $I^{clear}$, we first conduct *Erosion* ⊖ to remove background noise, then perform *Dilation* ⊕ twice to thicken the number lines, as described in Equation (2).

(2) $$I_{i\times j}^{clear}=\left(\left(I^{ori}⊖SE\right)⊕SE\right)⊕SE$$

where $I^{ori}$ and SE indicate an original image and a structuring element, respectively. The structural element SE is a matrix used to identify pixels in an image and define a neighborhood for processing each pixel. Here, we use a *square* structuring element whose width is 2 pixels.

As a rule of thumb, pre-processing of an input image is very important for digit localization, i.e., the generation of ROI regions for digits in the image. Poorly designed pre-processing leads to digit localization errors. For example, digit localization divides a single digit into two ROIs or treats multiple digits as a single ROI. Therefore, pre-processing should be appropriately adjusted according to the character of the image to be sensed.

#### 3.2.3. Blob Analysis

A blob is defined by connected pixels. The blob analysis in Matlab calculates statistics for labeled regions of a binary image and returns quantities such as the area, centroid, and bounding box. The area is the number of pixels in a labeled region, and the centroid is the center coordinate of each region. The bounding box returns an M×4 matrix, where *M* represents the number of blobs. Each row of the matrix consists of a four-element vector [x, y, width, height] in pixel coordinates.

We implemented the blob analysis function by using a blob analysis block in Matlab Simulink, and the output value of the block was also used in the next step to determine the final ROIs.

#### 3.2.4. ROI Detection

**Figure 3:**
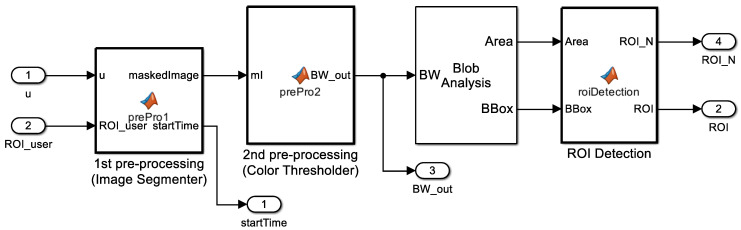
An ROI detection block in digit localization.

From the blob analysis block, we could get many bounding boxes where objects were expected. We could filter out unnecessary bounding boxes by using object information from the training dataset. Anchor boxes are a predefined set of bounding boxes with a specific height and width. Using anchor boxes significantly reduces computational cost because sliding windows compute separate predictions for every potential position. An ROI detection block captures the scale and aspect ratio of objects in an image, as shown in Figure 3. The block then filters out unnecessary bounding boxes that do not fit a predefined size of anchor boxes.

### 3.3. Digit Classification (ROMI Subsystem 2)

**Figure 4:**
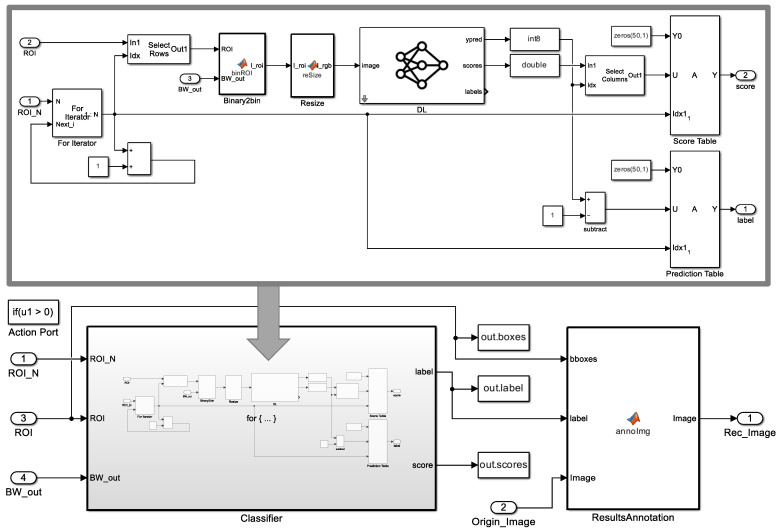
Classifier block in digit classification.

Digit recognition crops a binary image from each ROI and classifies the number. The number of digits can be zero or several, depending on the image. To enable digit recognition on multiple digit variables, we use thed *For iterator subsystem* block in Matlab Simulink. The iterative subsystem was run as many times as the number of digit variables on the LCD display. Figure 4 shows a classifier block in digit classification. Depending on the number of ROIs ROI_N, the classifier block performed the classification task iteratively. In the classifier block, the pre-processing cropped the input image to the ROI size in the Binary2bin block and then resized the cropped image to fit the input size of the deep learning model in the Resize block. The DL model predicted labels and predictive scores for the ROIs and sent them to the output. The ResultAnnotation block created a recognized image by annotating the original image with ROIs and labels.

A binary image cropped by ROIs went through three pre-processing steps before being used as an input image for the deep learning model, as follows.

#### 3.3.1. Zero Padding

The first step for deep learning was to make the image a square image. The input image of the deep learning model was of square shape, but the digit image was of rectangular shape. Converting a digit image into an input image for the deep learning model stretched or shrank the original image with a size i×j×3. To better preserve the shape of the original image, we used the zero-padding technique to generate a square image. Zero padding filled the sides, top, and bottom with zero values by *X* and *Y*.

(3) X=⌊(max([i,j])−i)/2⌋

(4) Y=⌊(max([i,j])−j)/2⌋

#### 3.3.2. Complemented Binary Image

We transformed the original image $I_{X\times Y\times 3}^{origin}$ into a gray-color image $I_{X\times Y\times 1}^{gray}$ and converted it into a binary image $I_{X\times Y\times 1}^{binary}$. We then used the complement of the binary image to make a complemented image. In the complement of the binary image, 0 became 1, and 1 became 0, i.e., black and white were inverted. We computed the complement of the binary image by using the *imcomplement* Matlab function. The complemented binary image $I_{X\times Y\times 1}^{com}$ was generated by using Equation (5).

(5) $$I_{X\times Y\times 1}^{com}=\left|I_{X\times Y\times 1}^{binary}-M_{X\times Y\times 1}^{one}\right|$$

where $M_{X\times Y\times 1}^{one}$ is a matrix of size X by Y, with all matrix values as elements of 1. The complemented binary image $I_{X\times Y\times 1}^{com}$ could be obtained by using the absolute value of the difference between the values of the $I_{X\times Y\times 1}^{binary}$ matrix and the $M_{X\times Y\times 1}^{one}$ matrix.

#### 3.3.3. Resizing

We used the DL models, i.e., ten pre-trained CNN models, for the image classifier in ROMI. Depending on the size *n* of the pre-trained CNN models, we needed to adjust the input size of the image. We carried out the resizing function by using the *imresize* Matlab function with the input size *n*. It returned a resized image $I_{n\times n\times 1}^{res}$ of size n×n×1 from the complemented binary image $I_{X\times Y\times 1}^{com}$ with size X×Y×1.

In addition, the pre-trained CNN model took RGB images as input images. Since the previously pre-processed image was one-dimensional image data, it needed to be converted into a three-dimensional image once more. We concatenated three identical one-dimensional images $I_{n\times n\times 1}^{res}$ to create a three-dimensional RGB image $I_{n\times n\times 3}^{rgb}$, as shown in Equation (6).

(6) $$I_{n\times n\times 3}^{rgb}=[I_{n\times n\times 1}^{res}|I_{n\times n\times 1}^{res}\left|I_{n\times n\times 1}^{res}\right]$$

#### 3.3.4. Image Classifier

To develop a DL model for digit recognition in ROMI, we use pre-trained CNN models in Matlab. Most of the pre-trained CNN models were trained using a subset of the ImageNet database and showed remarkable performance in the ImageNet Large-Scale Visual Recognition Challenge (ILSVRC) [52]. We used a transfer learning approach to re-train a pre-trained network that was already trained on a large dataset for our new dataset.

Among the pre-trained CNN models available in Matlab, we selected ten pre-trained CNN models for a DL classifier in ROMI as candidates by considering the depth, size, and parameters to be applied to the NVIDIA GPU embedded platforms. The selected DL models and model information are shown in Table 2.

The original pre-trained CNN models were trained to classify 1000 object categories, such as keyboards, coffee mugs, pencils, and many animals.

To develop the DL model in ROMI, we first found a learnable (fully connected or convolutional) layer in a pre-trained CNN model and added a new classification layer with ten classes from the digit 0 to 9. Then, we retrained the pre-trained CNN model by using our new training dataset.

We trained the model with the stochastic gradient descent with momentum (SGDM) optimizer. The parameters of the training deep learning neural network were set to 32 for the mini-batch size, 0.0003 for the initial learning rate, 3 for the validation frequency, and 100 for the maximum epoch.

### 3.4. Digit Annotation (ROMI Subsystem 3)

**Figure 5:**
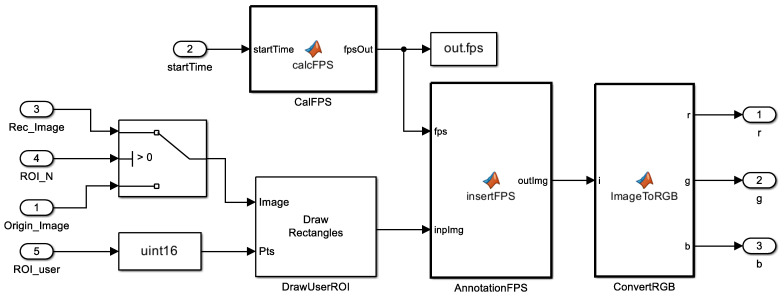
Digit annotation block in ROMI.

The last subsystem block in ROMI displayed the user ROI, digit recognition results, and frames per second (FPS), as shown in Figure 5. The user ROI was the region that the user wanted to recognize and was displayed as a solid black line. The image containing the result recognized by the digit classification was used as the input image for the digit annotation. If the image had no digits in it, i.e., the ROI was empty, the original image was used. The FPS value was used to evaluate the speed of digit recognition and estimate the computational performance by measuring the time from the beginning of the digit detector to the end of the digit recognizer, i.e., FPS.

### 3.5. Deployment on Embedded Hardware Platforms

The final step in developing the ROMI system was deploying the system on the target embedded platforms. We used three NVIDIA GPU embedded platforms: Jetson Nano, Jetson Xavier, and Jetson AGX Xavier. Matlab Simulink blocks and modules related to deep learning were optimized with the NVIDIA CUDA Deep Neural Network library (cuDNN) by optimizing the computation-intensive parts of deep learning networks. The Matlab GPU Coder then generated the GPU code, thus accelerating the computing performance on the target GPU embedded platforms.

We briefly introduce three NVIDIA GPU embedded platforms, as shown in Table 3. NVIDIA Jetson Nano is a compact, entry-level embedded platform commonly used for education or simple applications for applying simple deep learning models. NVIDIA Jetson Xavier NX is widely used in various Artificial Intelligence of Things (AIoT) systems that require adequate computing power, such as commercial robots, medical devices, and smart cameras. NVIDIA Jetson AGX is an embedded system that delivers the highest AI performance and is used in applications that require high-performance computing power, such as AI-powered autonomous machines.

## 4. Evaluations

This section first defines the performance metrics used to evaluate the system performance for ROMI. We then assess the system performance in terms of digit recognition and runtime.

### 4.1. Evaluation Setup

In this section, we describe two performance metrics. The first metric evaluates the recognition performance while considering both digit localization and classification. The second metric evaluates the operating speed, i.e., runtime performance, on the NVIDIA GPU embedded platforms.

#### 4.1.1. Evaluation Metric for Digit Recognition

Before describing the evaluation metrics, we clarify the terms object recognition and object detection to reduce the confusion about word usage. Object recognition is similar to object detection and is sometimes used interchangeably with it. However, object recognition is used to classify multiple classes, whereas object detection is used to detect a small class, such as in human and vehicle detection. In this work, we use the term digit recognition because we have ten classes from 0 to 9.

Object recognition is used as a term to describe both object localization and object classification. Object localization is finding the presence of objects in an image and drawing their location with a bounding box. Object classification is identifying the class within each bounding box of an image. That is, object recognition aims not only to localize spatially, but also to accurately classify objects within an image. The outputs of object recognition are one or more bounding boxes with a class label attached to each bounding box.

Average precision (AP) and mean average precision (mAP) are popular performance metrics for measuring the accuracy of object detectors [55,56]. When evaluating recognition performance, two metrics should be considered together: localization and classification of digits in an image. For example, we need to assess whether a predicted class is the true class and how close the predicted bounding box is to the ground-truth bounding box. A single performance metric considering the two metrics is AP for a single class and mAP for multiple classes. In this work, we used mAP as a performance metric for digit recognition. To calculate mAP, we need to understand a few of the following metrics.

The first step in calculating mAP is to find the degree of overlap between the ground-truth and predicted bounding boxes. The most common overlap criterion is the intersection over union (IoU).

IoU is a number that quantifies the ratio between the intersection and union areas of the predicted bounding box $BBox^{pre}$ and the ground-truth bounding box $BBox^{gro}$, as depicted in Equation (7).

(7) $$IoU=\frac{Area(BBox^{pre}\cap BBox^{gro})}{Area(BBox^{pre}\cup BBox^{gro})}$$

The IoU quantifies the closeness between the predicted bounding box and the ground-truth bounding box. If the two bounding boxes completely overlap, the value of IoU is 1, which means that the prediction is perfect. On the other hand, if the two bounding boxes do not overlap, the value of IoU is 0.

Based on the IoU and confidence threshold τ, the prediction result Result (Positive or Negative) is determined, as depicted in Equation (8).

(8) $$Result\left(IoU\right)=\begin{array}{cc} Positive, & IoU\geq \tau ,\\ Negative, & IoU<\tau . \end{array}$$

Using the ground-truth bounding box (true or false) and the prediction results (positive or negative), we can calculate the following four basic metrics: true positive (TP), false positive (FP), false negative (FN), and true negative (TN).

- TP: The model predicts that the predicted bounding box is where the ground-truth box is (positive), and the prediction is correct (true).
- FP: The model predicts that the predicted bounding box is where the ground-truth box is (positive), and the prediction is wrong (false).
- FN: The model predicts that the predicted bounding box is not where the ground-truth box is (negative), and the prediction is wrong (false).
- TN: The model predicts that the predicted bounding box is not where the ground-truth box is (negative), and the prediction is correct (true).

Based on these four basic metrics for each labeled class, we can calculate two performance metrics: precision and recall.

Precision tells how accurate the model is, i.e., how many correct predictions are in the total predictions, as depicted in Equation (9).

(9) $$Precision=\frac{TP}{TP+FP}$$

Recall tells how many correct predictions are in the total ground truth, as depicted in Equation (10).

(10) $$Recall=\frac{TP}{TP+FP}$$

By changing the values of the confidence threshold τ, a precision–recall curve can be obtained. The precision–recall curve shows the tradeoff between the precision and the recall for different thresholds τ. An ideal model shows high precision and high recall in both performance metrics.

The AP summarizes the precision–recall curve into a single value between 0 and 1. The AP is high when both the precision and the recall are high. If at least one of them is low in the confidence threshold τ, the AP is also low. The AP is calculated by measuring the area under the precision–recall curve, as depicted in Equation (11).

(11) $$AP=\int_{r=0}^{1}p\left(r\right)\;dr$$

where p(r) represents the precision values across the recall values *r* from 0 to 1. In actual calculations for the AP, approximation or interpolation methods are usually used to calculate the precision *p* at the confidence threshold τ, as depicted in Equation (12).

(12) $$AP=\sum_{\tau =0}^{\tau =n-1}\left[r\left(\tau \right)-r\left(\tau +1\right)\right]*p\left(\tau \right)$$

where *n* is the number of confidence thresholds τ. After calculating the AP value for each class, the mean average precision mAP is calculated by averaging AP across all classes, as depicted in Equation (13).

(13) $$mAP=\frac{1}{C}\sum_{k=1}^{C}AP\left(k\right)$$

where *C* and *k* indicate the total numbers of classes and indexes, respectively. In summary, the mAP quantifies the performance in object localization and object classification for object recognition in ROMI as a single metric.

#### 4.1.2. Evaluation Metric for Runtime Performance

For the evaluation of the runtime performance, we measured the execution speed on the embedded platforms by using the performance metric of FPS. FPS is a common metric for measuring graphic performance, such as in animation [57], object detection [58], and rendering [59].

We used the mean FPS (mFPS) as the metric for runtime evaluation, as depicted in Equation (14).

(14) $$mFPS=\sum_{k=1}^{n}FPS\left(k\right)$$

where *n* is the number of samples. An image file with a larger size is computationally expensive, so FPS decreases. We can improve FPS by simply reducing the image size, but there is a tradeoff between speed and accuracy.

This work measured the runtime performance (mFPS) in digit recognition on an LCD display. We captured the display with a Logitech StreamCam camera with VGA resolution (640 × 480 pixels). Note that current work focused on developing a digit recognition embedded system with VGA resolution and analyzing the FPS on various NVIDIA GPU platforms as a proof-of-concept study.

### 4.2. Digit Recognition Performance Evaluation

This section evaluates the digit recognition performance to select the best DL model in ROMI. We used ten pre-trained CNN models and retrained the models with a new dataset, i.e., we used transfer learning. We also evaluated the recognition performance through data augmentation.

#### 4.2.1. Data Augmentation for Training the DL Model

Deep learning requires large amounts of training data to train a model. However, collecting a training dataset takes much time and effort. To alleviate these difficulties, we used a data augmentation technique to regenerate samples from the original training data. Data augmentation is a technique for creating better deep learning models by increasing the size and quality of training data [60,61]. Data transformations, such as flipping, cropping, scaling, and rotating, were used to manipulate the training data in data augmentation. We regenerated the training data by using a Matlab function for image data augmentation imageDataAugmenter. This work only used a scaling factor (from 0.9 to 1), since ROMI could get closer to medical devices and capture frontal images on LCD displays.

In addition, DL models require relatively more data than ML models do, so a sufficient training dataset is essential for training DL models. To reduce the training data acquisition effort, we propose a simple data generation method that creates training datasets with a sufficient size by replicating the original training dataset. The original training dataset consisted of ten images for each digit. We expanded the training dataset and made an augmented training dataset through data augmentation. For example, the original training dataset was duplicated 10 to 100 times, and the duplicated dataset was used in image data augmentation (imageDataAugmenter) to generate new training data. That is, data augmentation randomly perturbed the original training dataset, resulting in a slightly different training dataset. We used the dataset that showed the best recognition performance for each model as the augmented training dataset candidate.

Table 4 shows the results from data augmentation in the DL models. Our approach was to effectively augment the training dataset for the DL models with a small dataset consisting of ten images per digit. The results showed that data augmentation effectively improved the recognition performance in all ten DL models. The digit recognition performance (mAP) was improved from a minimum of 1% to a maximum of 38% with data augmentation. The best DL model for digit recognition was alexnet, with the mAP value of 0.989.

In addition, we evaluated the recognition performance when we trained the DL models with public digit datasets that were freely available on the internet. We used two open digit datasets [62]: the Digits dataset and the Modified National Institute of Standards and Technology database (MNIST) dataset. The Digits dataset consisted of a total of 10,000 synthetic grayscale images of handwritten digits, with 1000 images for each digit from digit 0 to digit 9. The MNIST dataset consisted of more data than the Digits dataset. It consisted of a total of 70,000 handwritten digits and 7000 images for each digit from digit 0 to digit 9. When we used the two open digit datasets and trained the ten DL models, the recognition performance was poor, since they were basically handwritten digit datasets and were not effective for our application, i.e., seven-segment digit recognition. The MNIST dataset had seven times as many datasets as the Digits dataset and performsd relatively better.

#### 4.2.2. Trained DL Model Analysis

We retrained the ten pre-trained CNN models with our new digit dataset with ten classes, i.e., we used transfer learning. We used ten pre-trained CNN models that performed well in ILSVRC. Originally, they were trained by using the ImageNet dataset consisting of 14 million images with 1000 classes [52].

In the performance evaluation for digit recognition, relatively simple and trained CNN models, such as squeezenet, googlenet, and alexnet, showed excellent performance with mAP values of 0.976 or higher. Because the problem of digit recognition is simple, a relatively simple DL model seems to be suitable and shows good recognition performance.

Table 5 shows the recognition performance for each digit among the ten DL models in detail. The overall digit recognition was good in the ten DL models, but we confirmed that the recognition performance was relatively poor in digit 6, digit 8, and digit 9. The reason may have been that the digits with circles were similar in shape, and the poor DL models could not tell the difference between the top and bottom of the image. The alexnet model showed the best recognition performance among the ten DL models, with a minimum of 0.991 for the AP and a maximum of 0.993 for the AP for the ten digits from 0 to 9.

**Figure 6:**
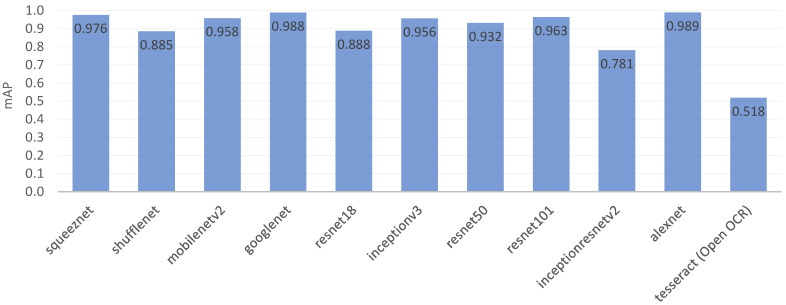
Recognition performance evaluation results for the trained DL models.

We further compared the recognition performance when using the ten trained DL models and an open OCR algorithm, i.e., the tesseract OCR. The Matlab function ocr works based on the tesseract engine, and we could use the trained tesseract OCR model by setting the language to ‘seven-segment’. The trained tesseract OCR model showed 0.518 for the mAP, which was not as good as the other DL models. Figure 6 summarizes the recognition performance of the eleven trained OCR models. One of the reasons for the low performance of the tesseract OCR model is that the model was not trained with our dataset. There may have been a difference between the dataset used by the tesseract OCR and our dataset. Note that retraining or fine-tuning of the tesseract OCR was not within the scope of this work. The tesseract OCR model did not work on the Matlab Simulink platform, so it was excluded from the OCR model of ROMI.

### 4.3. Runtime Performance Evaluation

We deployed the trained models on various embedded platforms by using the cuDNN GPU coder. This work used three NVIDIA GPU platforms: NVIDIA Jetson Nano, NVIDIA Jetson Xavier NX, and NVIDIA AGX Xavier. We then evaluated the runtime performance (mFPS) given two considerations: three NVIDIA GPU platforms and ten pre-trained CNN model architectures.

#### 4.3.1. NVIDIA Jetson GPU Platforms

We deployed trained DL models on NVIDIA Jetson GPU platforms: Jetson Nano, Xavier NX, and AGX Xavier. The executable code of ROMI was generated by using Matlab GPU Coder. Matlab GPU Coder generated the executable code by using the NVIDIA CUDA deep neural network library (cuDNN) for NVIDIA GPUs. The generated code allowed the Matlab function blocks to run fast on NVIDIA GPUs.

Table 6 shows the runtime performance of the ten DL models depending on three different NVIDIA GPU platforms. The results show that the runtime performance of the ten DL models improved with better GPU platforms. In the benchmarks of the NVIDIA GPUs, the AI performance was 472 GFLOPs on the Jetson Nano, 21 TOPs on the Xavier NX, and 32 TOPs on the AGX Xavier, respectively. In addition, lightweight model architectures, such as squeeznet and shufflenet, showed fast runtime performance. On the other hand, heavy model architectures, such resnet101 and inceptionresnetv2, had slow runtime performance.

#### 4.3.2. DL Model Deployment

Embedded platforms have limited resources available, so we needed to check the actual runtime performance of the DL models to reduce system errors or failures. We used the ten pre-trained CNN models with different architectures. The models were improved to enhance the classification performance of the ImageNet dataset by modifying the architecture of the model, such as the layer type, size, width, and depth.

**Figure 7:**
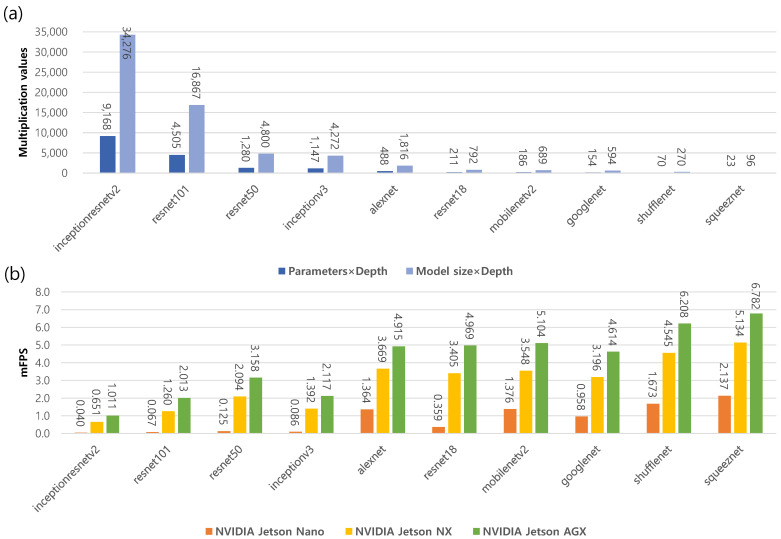
Runtime performance evaluation considering the DL models and NVIDIA GPU platforms. (**a**) Multiplication values for the ten DL models. (**b**) The mFPS of the NVIDIA GPU platforms with the ten DL models.

Figure 7 shows the runtime performance of the ten DL models on the three different NVIDIA GPU platforms. As the number of learnable parameters in a DL model increased, the size of the model increased proportionally. On the other hand, there were model architectures where the model size and parameters were large, but the depth was small, e.g., alexnet. In our observation, the runtime performance was affected by multiple factors, rather than a single factor. We sorted the products of two factors in descending order, i.e., parameter × depth and model size × depth, as shown in Figure 7a. The experimental results show that the runtime performance (mFPS) increased linearly, except for that of inceptionv3 and googlenet, as shown in Figure 7b. Exceptional cases may arise from the unique architecture of each model, and we experimentally showed an inverse relationship between the multiplicative value and mFPS in the general case.

## 5. Discussion

To improve care for ICU patients, we propose an embedded system called ROMI that can read digits from LCD screens on medical devices. In this section, we address the limitations of our system and future work.

There are some limitations in this work. First, a vision-based OCR approach is highly susceptible to camera artifacts, such as the angle, distance between an object and a camera, tilt, camera focus, light reflection, and brightness. In this work, we did not consider these artifacts that affected the quality of the input images. These artifacts can be mitigated to some extent by using a robotic system consisting of a mobile robot and a robotic arm. Second, we evaluated the system performance using only the LCD of an Arduino, so the testing phase was limited. Generating digits from actual medical devices is difficult and requires reverse engineering to control the device. We used an Arduino device with an LCD module as a proof-of-concept device. This device allowed the user to output the desired digits on the LCD. Using the Arduino, we could effectively develop the entire developmental process: data collection, DL model development, and deployment of embedded systems. Note that we focused on the general developmental process for digit recognition as a proof-of-concept study.

In future work, we will explore additional techniques, such as DL model quantization and mobile robot control, to enhance the system performance of ROMI and make it work automatically. First, runtime performance can be further improved by quantizing DL models in the target embedded system. DL models consist of many processing layers, including convolutional layers. Most pre-trained CNNs use single-precision floating-point data types, so even small networks require significant amounts of memory and hardware. Quantization restricts data types to 8-bit scaled integer data types, so the models are computationally less powerful and require fewer memory resources [63]. We will research further quantization by using TensorRT, a high-performance inference library in NVIDIA GPU platforms. Second, ROMI is a mobile robot that moves to medical devices and recognizes digits on their displays in ICUs. Therefore, it is necessary to develop the basic control functions for a mobile robot: navigation, localization, and obstacle avoidance [64]. Developing the control of a robot arm is also required to capture the displays well. The ROMI system was developed with Matlab Simulink. The key functional block, ROMI, for digit recognition was developed and implemented as a proof-of-concept study. ROMI will be supplemented by adding robot control blocks to make it practical in ICUs.

## 6. Conclusions

Monitoring critically ill patients is essential because their conditions might suddenly worsen, requiring timely and appropriate treatment to save lives. However, due to limited human resources, there are real limitations in responding appropriately to disasters, such as COVID-19. In addition, not all medical devices are monitored by ICUs’ control centers because some medical devices do not have communication capabilities. To address these practical problems, ROMI is proposed to monitor critically ill patients on behalf of medical staff in the form of non-face-to-face monitoring.

We used Matlab Simulink to build ROMI for digit recognition. ROMI consists of three main subsystems: a digit detector, a digit classifier, and digit annotation. We retrained ten pre-trained CNN models by using the transfer learning technique and selected the model with the highest performance in our dataset. The best DL model, i.e., alexnet, showed high performance in digit recognition with 0.989 for the mAP. We also evaluated dependence of the runtime performance on NVIDIA Jetson GPU platforms: Jetson Nano, Xavier NX, and AGX Xavier. The evaluation results showed that the runtime performance, i.e., mFPS, was affected by model complexity in relation to depth, the number of parameters, and the model size. The runtime performance results will be used to design the ideal hardware-embedded ROMI system.

As a proof-of-concept study, we built a key function block, ROMI, that recognizes digits displayed on the LCD screens of medical devices. ROMI is mounted on a robotic system and helps keep an eye on ICU patients in real time. We hope that ROMI will play an essential role in effectively helping medical staff and reducing patient mortality.

## Figures and Tables

**Table 1:** Related work on monitoring and integrating medical data.

Method	Target Medical Device	Solution/Approach	Problem/Challenge
CodeBlue [40]	A ventilator and ECG	MICA2 motes	Limited data logging
OpenICE [41]	Bedside medical devices	BeagleBone single-board computers	Limited data logging
AlarmNet [42]	Heart rate, pulse oximetry, ECG, body movement	MicaZ and Telos Sky motes	Limited data logging
MEDiSN [43]	Various vital signs, such as pulse oximetry and pulse rate	miTag motes	Limited data logging
VentConnect [46]	Ventilators	Capture device via a display interface converter	Ventilator only
PACMAN [47]	Pulse oximeter	Digit OCR of images taken with smartphone cameras	Pulse oximeter only and model calibration
ROMI (Ours)	Multiple ICU medical devices	Medical device digit OCR using a robotic system	Model calibration

**Table 2:** Network information of the ten pre-trained CNN models [53].

Network	Depth	Size [MB]	Parameters (Millions)	Input Size
squeeznet	18	5.2	1.24	227 × 227 × 3
shufflenet	50	5.4	1.4	224 × 224 × 3
mobilenetv2	53	13	3.5	224 × 224 × 3
googlenet	22	27	7.0	224 × 224 × 3
resnet18	18	44	11.7	224 × 224 × 3
inceptionv3	48	89	23.9	299 × 299 × 3
resnet50	50	96	25.6	224 × 224 × 3
resnet101	101	167	44.6	224 × 224 × 3
inceptionresnetv2	164	209	55.9	299 × 299 × 3
alexnet	8	227	61.0	227 × 227 × 3

**Table 3:** NVIDIA GPU embedded platforms [54].

DL Model	Jetson Nano	Jetson Xavier NX	Jetson AGX Xavier
GPU	128-core Maxwell	384-core NVIDIA Volta™ GPU with 48 Tensor Cores	NVIDIA Volta architecture with 512 NVIDIA CUDA cores and 64 Tensor cores
AI Performance	472 GFLOPs	21 TOPs	32 TOPs
CPU	Quad-core ARM A57 @ 1.43 GHz	6-core NVIDIA Carmel ARM^®^ v8.2 64-bit CPU 6 MB L2 + 4 MB L3	8-core NVIDIA Carmel Armv8.2 64-bit CPU 8 MB L2 + 4 MB L3
Memory	4 GB 64-bit LPDDR4 25.6 GB/s @ 1.43 GHz	8 GB 128-bit LPDDR4x 59.7 GB/s	32 GB 256-bit LPDDR4x 136.5 GB/s
Storage	microSD	16 GB eMMC 5.1	32 GB eMMC 5.1
Power	5 W|10 W	10 W|15 W|20 W	310 W|15 W|30 W

GFLOPs: GPU floating-point operations per second; TOPs: tera-operations per second.

**Table 4:** Recognition performance (mAP) evaluation for training DL models depending on the dataset.

DL Model	Ten Images without DA	Ten Images with DA (Ours)	Digits Dataset (Open Dataset)	MNIST Dataset (Open Dataset)
squeeznet	0.790	0.976	0.073	0.397
shufflenet	0.530	0.885	0.013	0.158
mobilenetv2	0.579	0.958	0.010	0.264
googlenet	0.964	0.988	0.153	0.535
resnet18	0.540	0.888	0.097	0.104
inceptionv3	0.754	0.956	0.011	0.072
resnet50	0.680	0.932	0.015	0.133
resnet101	0.830	0.963	0.011	0.198
inceptionresnetv2	0.329	0.781	0.014	0.010
alexnet	0.984	0.989	0.138	0.489

DA: data augmentation.

**Table 5:** Recognition performance (mAP) evaluation for evaluating the trained DL models.

OCR Model	Digit 0	Digit 1	Digit 2	Digit 3	Digit 4	Digit 5	Digit 6	Digit 7	Digit 8	Digit 9	Total
squeeznet	0.985	0.989	0.988	0.992	0.991	0.935	0.992	0.991	0.982	0.914	0.976
shufflenet	0.775	0.990	0.633	0.910	0.991	0.898	0.918	0.874	0.952	0.908	0.885
mobilenetv2	0.985	0.990	0.987	0.990	0.990	0.980	0.822	0.976	0.911	0.947	0.958
googlenet	0.986	0.990	0.989	0.992	0.991	0.991	0.993	0.992	0.984	0.977	0.988
resnet18	0.985	0.990	0.989	0.893	0.991	0.988	0.693	0.985	0.739	0.627	0.888
inceptionv3	0.984	0.990	0.967	0.973	0.988	0.922	0.944	0.991	0.934	0.869	0.956
resnet50	0.968	0.990	0.984	0.977	0.990	0.973	0.869	0.992	0.939	0.634	0.932
resnet101	0.986	0.990	0.981	0.898	0.974	0.991	0.992	0.992	0.842	0.984	0.963
inceptionresnetv2	0.944	0.987	0.504	0.988	0.858	0.890	0.528	0.949	0.556	0.606	0.781
alexnet	0.986	0.990	0.989	0.992	0.991	0.991	0.993	0.992	0.984	0.985	0.989
tesseract (Open OCR)	0.129	0.978	0.445	0.252	0.006	0.700	0.902	0.484	0.490	0.795	0.518

**Table 6:** Runtime performance (mFPS) evaluation depending on embedded hardware platforms.

DL Model	NVIDIA Jetson Nano	NVIDIA Jetson Xavier NX	NVIDIA Jetson AGX Xavier
squeeznet	2.147	5.134	6.782
shufflenet	1.673	4.545	6.208
mobilenetv2	1.376	3.548	5.104
googlenet	0.958	3.196	4.614
resnet18	0.359	3.405	4.969
inceptionv3	0.086	1.392	2.117
resnet50	0.125	2.094	3.158
resnet101	0.067	1.260	2.013
inceptionresnetv2	0.040	0.651	1.011
alexnet	1.364	3.669	4.915

## Data Availability

Not applicable.

## Abbreviations

The following abbreviations are used in this manuscript:

ICUs	Intensive Care Units
EMR	Electronic Medical Record
HL7	Health Level 7
IoT	Internet of Things
OCR	Optical Characteristic Recognition
CNN	Convolutional Neural Network
DL	Deep Learning
GPU	Graphics Processing Unit
MSER	Maximally Stable Extremal Regions
MDD	Medical Device Dongle
SpO2	Oxygen saturation
ECG	Electrocardiogram
LCD	Liquid Crystal Display
ML	Machine Learning
SVM	Support Vector Machine
HOG	Histogram of Oriented Gradients
ROI	Region of Interest
HSV	Hue, Saturation, and Value
ILSVRC	ImageNet Large-Scale Visual Recognition Challenge
SGDM	Stochastic Gradient Descent with Momentum
FPS	Frames per Second
mFPS	Mean Frames per Second
cuDNN	NVIDIA CUDA Library
AIoT	Artificial Intelligence of Things
GFLOPs	GPU Floating-Point Operations Per Second
TOPs	Tera-Operations per Second
AP	Average Precision
mAP	Mean Average Precision
MNIST	Modified National Institute of Standards and Technology database
IoU	Intersection over Union
TP	True Positive
FP	False Positive
FN	False Negative
TN	True Negative
